# Hereditary Insanity

**Published:** 1850-06

**Authors:** 


Hereditary Insanity.—M. Baillarger is engaged on a series of "anatomical, physiological, and pathological researches upon the nervous system," but he has not yet completed the work, nor, in the present state of psychological science at Paris, is there much prospect of it ; a translation into English, doubtless, would be eagerly read by the profession at large. His observations on the development of the brain form a remarkable addition to the knowledge we have obtained through Reil, Teidmann, and Desmoulins ; he arrives at the conclusion that this organ is developed from within, and is increased by introsusception. His observations upon the hereditary nature of madness are invaluable ; they are the result of a long series of indefatigable labors, and show how much he is in earnest in his inquiries after facts. He chose the three following questions for solution :

Is the madness of the mother more frequently transmitted to the child than that of the father ?

In the case of hereditary madness, is the disease of the mother transmitted to a greater number of children than a similar malady in the father ?

Is madness more often transmitted from the mother to the daughter—and from the father to the sons ?

No less than 600 cases have been investigated by him for the purpose of arriving at some distinct conclusions. It would be difficult for any one but an individual so advantageously placed as is Dr. Baillarger, at the Bicetre, to have been able to collect such a quantity of statistic details as to have furnished him with proper data, for there are no documents existing which could be of much service ; the only report of the kind being one which was made by Aubonel and Thore, at the Bicetre, and this was of so limited a nature ar to be of comparatively little value. From Dr. Baillârger's cases the result was, that of 453 insane individuals, 271 had become so through the mother, if such an expression may be allowed, and 182 through the father, answering the first question by a large proportion passing through the female side.

The second question was resolved from 271 families, in which the mother had transmitted the disease—in one infant in 203 cases ; in two infants 62 ; in three, five cases ; in four infants, one ; that is to say, that in one-fonrth of the instances the mother's disease was shown in more than one case ; out of 182 families in which the disease descended from the father, a single child was diseased in 152 cases ; two children, 36 times ; three infants, four times ; that is to say, that the madness appeared only in one-sixth of the instances, proving that the mother's malady affected the greater number of the descendants.

The third question, whether the mother more frequently transmitred the disease to the girls, and the father to the sons, out of 346 children, he found that from the mother's side 197 girls, and 119 boys were affected ; and from 215, where the father had been the original source, 128 boys and 87 girls were affected, showing that the madness of the mother more frequently exhibited itself in the females than in the males ; whilst, on the contrary, the madness cf the father showed itself in the proportion of a third in the boys over the girls. Many are the physiological deductions which Dr. Baillarger has drawn from these inquiries, which, when the whole of his invaluable work is finished, will throw a great light upon the subject of hereditary madness.—*Journal of Insanity.*

